# Association between brominated flame retardants and periodontitis: a large-scale population-based study

**DOI:** 10.3389/fpubh.2024.1476953

**Published:** 2024-12-18

**Authors:** Hao Jiang, Jingwen Yin, Meixiang Wang, Aili Yuan, Jing Wu, Yi Lu

**Affiliations:** The Affiliated Taizhou People’s Hospital of Nanjing Medical University, Taizhou School of Clinical Medicine, Nanjing Medical University, Taizhou, China

**Keywords:** brominated flame retardants, periodontitis, WQS, QGC, NHANES

## Abstract

**Background:**

The association between brominated flame retardants (BFRs) and periodontitis has remained unclear.

**Methods:**

This research included adult participants from NHANES cycles 2009–2014. Survey-weighted generalized linear regressions were used to explore the associations between BFR exposure and periodontitis. Ln-transformed BFRs were treated as quantitative variables and then divided into four quartiles for qualitative analysis. Restricted cubic splines (RCSs) were utilized to investigate potential nonlinear relationships. Quantile weighted quantile sum (WQS) regression and quantile g-computation (QGC) analysis were performed to assess the overall effect of BFRs on periodontitis.

**Results:**

A total of 2,445 participants were included in this study. In the fully adjusted model, several ln-transformed BFR components were positively correlated with periodontitis: serum PBDE28, PBDE47, PBDE85, PBDE99, PBDE100, PBDE154, and PBB153. When expressed in quartiles, PBDE28, PBDE85, PBDE100, PBDE154, and PBB153 showed increased odds with periodontitis. We found significant nonlinear correlation between PBDE28, PBDE47, PBDE85, PBDE100, PBDE154, and PBB153 with periodontitis in the RCS regression. The WQS index for mixed BFR exposure was positively associated with periodontitis prevalence (OR: 1.52; 95% CI: 1.30–1.79, *p* < 0.001). Similarly, the QGC analysis showed a positive association between mixed BFR exposure and periodontitis prevalence (*β*: 1.29; 95% CI: 1.24–1.36, *p* < 0.001).

**Conclusion:**

This study indicates that overall exposure to BFRs is positively associated with the prevalence of periodontitis. Further research is needed to investigate the causal relationship and underlying mechanisms between BFRs and periodontitis.

## 1 Introduction

Brominated flame retardants (BFRs) hold a significant market share in the realm of flame retardants due to their low cost and high performance (1). There are over 75 commercially recognized BFRs that are utilized in a wide range of industries, including furniture, electronics, construction materials, automotive components, and more (2). In 1973, the substitution of magnesium oxide in cattle feed with commercial BFR Firemaster BP-6 in Michigan resulted in livestock losses, long-term health implications, and economic turmoil (3). The active chemical compound in Firemaster BP-6 is polybrominated biphenyl (PBB), which was later banned. This led to a significant increase in the use of polybrominated diphenyl ethers (PBDEs) in consumer products. Due to the structural similarities between PBDEs and PBBs, they share similar behavioral characteristics (4). PBDEs were subsequently acknowledged as persistent organic pollutants (POPs) under the Stockholm Convention in 2010.

Although several conventional BFRs, including PBBs and PBDEs, have been banned or restricted because of their established toxicity to both humans and wildlife, human exposure continues due to their environmental persistence and bioaccumulative properties (5). In addition, BFRs are prone to volatilization and degradation from the existence of large stockpiles and increased recycling of products containing BFRs, leading to human exposure through dietary intake, mother-to-child transmission, product use, and indoor dust, among other pathways (6). Previous studies have indicated that BFRs pose significant threats to human health through various toxicities, including endocrine disruption, reproductive toxicity, behavior effects, hepatotoxicity, neurotoxicity, immunotoxicity and developmental toxicity (7–10).

Periodontitis is a condition caused by dysbiosis in the microbial community of periodontal tissues. It disrupts the integrity of the tissues supporting the teeth through a complex interaction between periodontal pathogens and the host’s immune response (11). Roughly half of American adults are affected by periodontal disease, especially those with low income and the older adults (12–14). On a global scale, the prevalence of severe periodontal disease reaches 11%, with discomfort tooth mobility or tooth loss being experienced by individuals during a normal lifetime (15). This poses a significant public health challenge for our aging population. The early concept of a straight-line progression from gingivitis or pulpitis to marginal and apical periodontitis has been replaced by a highly complex understanding of the etiopathogenesis of periodontal diseases (13). The active infection of herpesvirus, specific bacterial species, and destructive immune responses are closely associated with the occurrence of periodontitis (16, 17). Furthermore, various environmental/risk factors such as genetic factors, aging, nutritional deficiencies, hormonal imbalances, and smoking interact to increase the risk of developing the condition (18).

Several studies have suggested that BFRs can disrupt calcium homeostasis and promote inflammation and oxidative stress, which are closely associated with the occurrence of periodontal disease (9, 18–20). Furthermore, not only individual BFRs have been shown to affect human health, but exposure to BFR mixtures is also positively correlated with cardiovascular diseases, COPD, liver function, non-alcoholic fatty liver disease, metabolic syndrome, and other diseases (6, 21, 22). However, there are still no studies investigating the effects of single or combined BFRs on periodontal disease. This study aims to address this gap by utilizing data from the NHANES database and employing various methodologies such as weighted logistic regression, RCS, WQS, and QGC. The ultimate objective is to heighten awareness and vigilance regarding the potential hazards associated with BFRs.

## 2 Methods

### 2.1 Study design and population

The National Health and Nutrition Examination Survey (NHANES) is a nationwide US survey assessing the health and nutrition of children and adults. Using multistage probability sampling, it collects data through interviews, physical exams, and lab tests every 2 years. The NHANES procedures are approved by the ethics committee of the National Center for Health Statistics (NCHS), with written consent from all adult participants. Further data analysis follows NCHS guidelines (23). The official website^1^ provides free access to NHANES.

**Figure 1:**
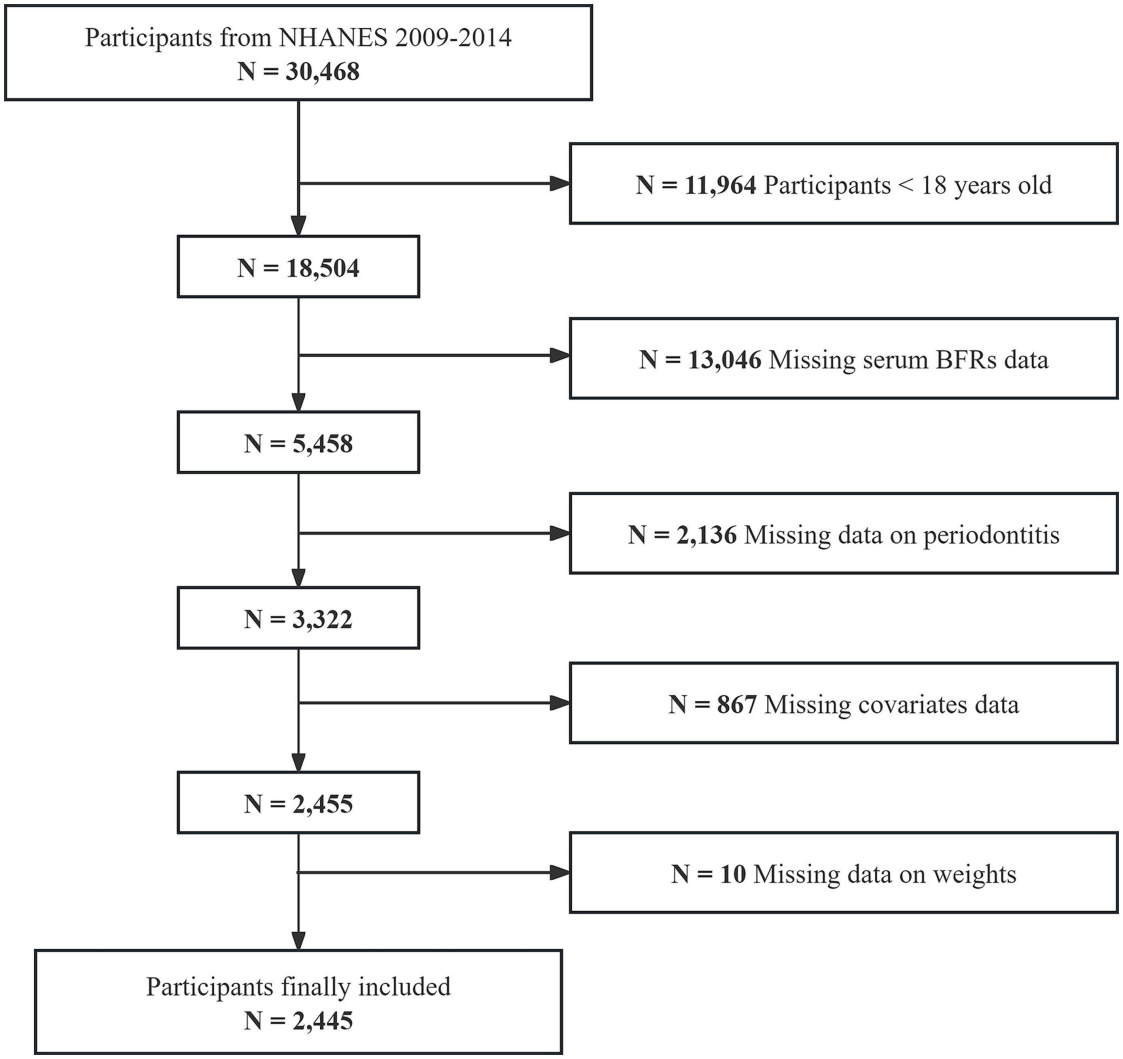
Flowchart of participants selection.

The initial sample for this study consisted of 30,468 individuals enrolled in three consecutive NHANES cycles from 2009 to 2014. Participants younger than 18 years old (*n* = 11,964) were excluded first. Then, those missing serum BFRs data (*n* = 13,046), periodontitis information (*n* = 2,136), covariates data (*n* = 867), and weight information (*n* = 10) were excluded. Ultimately, 2,445 individuals were included in the study (Figure 1).

### 2.2 Serum brominated flame retardants

The NHANES Laboratory Procedures Manual provides detailed instructions for the collection, storage, and processing of blood specimens. In the NHANES database, 12 different BFRs are quantified using automated liquid–liquid extraction and sample purification methods. These BFRs include PBDE17, PBDE28, PBDE47, PBDE66, PBDE85, PBDE99, PBDE100, PBDE153, PBDE154, PBDE183, PBDE209, and PBB153. Quantitative analysis is conducted using Isotope Dilution High-Resolution Gas Chromatography/Mass Spectrometry (GC/IDHRMS), as described by Johnson et al. (23). When levels fell below the detection limit, they were imputed as the detection limit divided by the square root of 2, following established protocols. To ensure the robustness of our study, we focused on nine BFRs with detection rates exceeding 70%, namely PBDE28, PBDE47, PBDE85, PBDE99, PBDE100, PBDE153, PBDE154, PBDE209, and PBB153 (22).

### 2.3 Periodontitis diagnosis

Periodontitis diagnosis relied on measurements of periodontal pocket probing depth (PD) and attachment loss (AL). Following CDC criteria (24), mild periodontitis was defined as having two or more interproximal sites with AL ≥ 3 mm and two or more interproximal sites with PD ≥ 4 mm (not on the same tooth), or one interproximal site with PD ≥ 5 mm. Moderate periodontitis was diagnosed when two or more interproximal sites had AL ≥ 4 mm or two or more interproximal sites had PD ≥ 5 mm, but not on the same tooth. Severe periodontitis was diagnosed when two or more interproximal sites had AL ≥ 6 mm (not on the same tooth), and at least one interproximal site had PD ≥ 5 mm. Patients not meeting any of these conditions were diagnosed with “no periodontitis.” The primary outcome of interest was the presence of moderate or severe periodontitis, while all other cases were categorized into the reference group.

### 2.4 Assessment of covariates

Socio-demographic factors, physical examination results, and health-related factors that might confound the correlation between BFRs and periodontitis were considered covariates. The covariates in the study included age, gender (male or female), race/ethnicity (non-Hispanic Black, non-Hispanic White, other Hispanic, Mexican American, or other race), education level (less than high school, high school diploma, or more than high school), body mass index (BMI), total energy intake, smoking status, alcohol use (yes or no), family poverty-to-income ratio (PIR), sleep trouble (yes or no), diabetes mellitus (yes or no), hyperlipidemia (yes or no), and hypertension (yes or no).

BMI was calculated as weight (kg) divided by height (m^2^). Individuals were classified as having normal BMI (<25), overweight (25.0–29.9), or obese (≥30). Total energy intake was obtained from a 24-h food recall. For categorization, low intake was defined as males consuming less than 2,000 kcal/day and females consuming less than 1,600 kcal/day. Adequate intake was considered for males consuming between 2,000 and 3,000 kcal/day and females consuming between 1,600 and 2,400 kcal/day. High intake was determined for males consuming over 3,000 kcal/day and females consuming over 2,400 kcal/day. Smoking status was categorized into three groups: never use, former use, and current use, based on two questions: (a) “Have you smoked at least 100 cigarettes in your entire life?” (SMQ020), and (b) “Do you now smoke cigarettes?” (SMQ935). Never use was identified as having smoked fewer than 100 cigarettes in their entire life. Former use was identified as having smoked at least 100 cigarettes in their entire life, but not currently smoking. Current use was identified as having smoked at least 100 cigarettes in their entire life, and currently smoking. Alcohol use was defined as consuming 12 or more alcoholic drinks in 1 year. PIR was categorized as follows: ≤1.30 for low-income, 1.31–3.50 for middle-income, and >3.50 for high-income. Sleep trouble was assessed through the question, “Have you reported to a healthcare provider that you experience difficulty sleeping?” (SLQ050). Responses included “Yes,” “No,” “Refused,” and “Do not know.” Participants who answered “Yes” were classified as having sleep trouble, while “Refused” and “Do not know” responses were considered missing data. Diabetes mellitus was defined by any of the following: physician-diagnosed diabetes, hemoglobin A1c (HbA1c) ≥ 6.5%, fasting blood glucose ≥7.0 mmol/L, 2-h oral glucose tolerance test (OGTT) blood glucose ≥11.1 mmol/L, or the use of prescribed diabetes medication or insulin. When any of these conditions were met, hyperlipidemia was diagnosed: triglyceride levels ≥150 mg/dL, total cholesterol levels ≥200 mg/dL, LDL cholesterol levels ≥130 mg/dL, HDL cholesterol levels <40 mg/dL for men or <50 mg/dL for women, or if the person was on lipid-lowering medications. Hypertension was determined based on in-home interviews and blood pressure measurements taken at the mobile examination center (MEC). Participants were considered to have hypertension if they met any of the following criteria: (1) Average systolic blood pressure ≥ 140 mmHg; (2) Average diastolic blood pressure ≥ 90 mmHg; (3) Self-reported hypertension; and (4) Ever prescribed anti-hypertensive medications (25).

### 2.5 Statistical analysis

The NHANES data analysis followed rigorous statistical procedures, adhering to NHANES weighting recommendations. Weighted analyses were conducted utilizing the 2-year examination weight (WTMEC2YR), primary sampling units (SDMVPSU), and strata (SDMVSTRA). Continuous variables with normal distribution were presented using means ± standard errors (SE), while variables with non-normal distribution were presented using medians and interquartile ranges (IQR). Categorical variables were expressed as numbers and proportions. T tests and Kruskal–Wallis H tests were used to test differences for continuous parameters. Pearson’s chi-squared test for categorical variables. Considering the right-skewed distributions of exposures, serum BFRs concentrations were ln-transformed. Spearman’s correlation coefficients were used to examine correlations between each pair of ln-transformed BFRs. Survey-weighted generalized linear regressions were conducted to investigate the associations between BFRs exposure and periodontitis. Ln-transformed BFRs served as quantitative variables and were then divided into four quartile groups to be used as qualitative variables. Three models were constructed: Model 1, the crude model, was not adjusted. Model 2 was adjusted for age, gender, race, PIR, education levels, BMI, energy intake, smoking status and alcohol use. Model 3, built upon Model 2, included adjustments for sleep trouble, hypertension, diabetes and hyperlipidemia. To investigate the potential nonlinear relationship between BFRs and periodontitis, we conducted a restricted cubic spline (RCS) analysis in the fully adjusted model. Finally, we employed quantile weighted quantile sum (WQS) regression and quantile g-computation (QGC) analysis to examine the overall effect of BFRs on periodontitis after adjusting for confounders. WQS regression constructs a weighted index to test associations in both directions (26). In this study, the data were randomly divided into a training set (40%) and a validation set (60%). An individual weight exceeding 1/9 (since there were 9 chemical components in the study) was considered above the threshold. QGC analysis uniformly integrates effects without directional constraints and is a straightforward method for estimating the coefficients of both individual and combined exposure variables on outcome variables (27).

Statistical analyses were completed by R software (version 4.3.2). *p* value <0.05 on two sides was seen as statistically significant.

## 3 Results

### 3.1 Baseline of the participants

A total of 2,445 participants were ultimately included in this study. Table 1 displays the baseline characteristics of these participants. The average age of the participants was 51 years old, with 48.85% being male. Significant differences were observed between the periodontitis group and the control group in terms of age, race, education levels, energy intake, PIR, smoking status, DM, and hypertension. However, the data did not reveal any significant differences between these two groups in terms of gender, BMI, alcohol consumption, sleep trouble, or hyperlipidemia. Additionally, periodontitis patients were found to have a higher likelihood of exposure to elevated levels of various BFRs, including PBDE28, PBDE47, PBDE85, PBDE99, PBDE100, PBDE154, PBDE209, and PBB153, based on observations.

**Table 1:** Baseline characteristics of the participants.

Characteristics	Total	Periodontitis	Non-periodontitis	*p* value
Age (SE)	51.37 (0.42)	55.38 (0.55)	48.10 (0.61)	**<0.001**
Gender, %				0.070
Male	1,189 (48.85)	727 (56.64)	462 (42.50)	
Female	1,256 (51.15)	556 (43.37)	700 (57.50)	
Race, %				**<0.001**
Mexican American	334 (7.29)	218 (10.11)	116 (4.98)	
Non-Hispanic Black	498 (10.74)	298 (12.97)	200 (8.91)	
Non-Hispanic White	1,134 (71.43)	524 (65.34)	610 (76.40)	
Other Hispanic	232 (4.79)	129 (5.28)	103 (4.39)	
Other Race	247 (5.76)	114 (6.30)	133 (5.33)	
Education, %				**<0.001**
High school	885 (31.60)	564 (41.00)	321 (23.93)	
Less than high school	179 (3.83)	135 (6.36)	44 (1.76)	
More than high school	1,381 (64.57)	584 (52.64)	797 (74.32)	
BMI, %				0.869
Normal	617 (26.02)	324 (25.29)	293 (26.61)	
Obese	989 (39.67)	515 (39.85)	474 (39.53)	
Overweight	839 (34.31)	444 (34.86)	395 (33.87)	
Energy intake, %				**0.027**
Low	973 (34.84)	574 (38.39)	399 (31.94)	
Adequate	1,076 (47.06)	509 (42.78)	567 (50.55)	
High	396 (18.11)	200 (18.83)	196 (17.51)	
PIR, %				**<0.001**
High income	874 (46.60)	363 (37.42)	511 (54.10)	
Low income	667 (17.22)	433 (23.07)	234 (12.44)	
Middle income	904 (36.18)	487 (39.51)	417 (33.46)	
Smoke, %				**<0.001**
Never	1,361 (56.21)	608 (46.77)	753 (63.93)	
Former	666 (27.25)	390 (30.48)	276 (24.62)	
Now	418 (16.54)	285 (22.76)	133 (11.45)	
Alcohol, %				0.884
Yes	2,149 (91.13)	1,122 (91.01)	1,027 (91.23)	
No	296 (8.87)	161 (8.99)	135 (8.77)	
Sleep trouble, %				0.726
Yes	664 (27.86)	340 (27.44)	324 (28.21)	
No	1,781 (72.14)	943 (72.56)	838 (71.79)	
DM, %				**0.009**
Yes	501 (16.01)	320 (18.86)	181 (13.68)	
No	1,944 (83.99)	963 (81.14)	981 (86.32)	
Hypertension, %				**0.001**
Yes	1,121 (41.39)	671 (47.05)	450 (36.76)	
No	1,324 (58.61)	612 (52.95)	712 (63.24)	
Hyperlipidemia, %				0.151
Yes	1,887 (76.40)	1,021 (77.96)	866 (75.12)	
No	558 (23.60)	262 (22.04)	296 (24.88)	
PBDE28	6.58 (4.74, 9.74)	7.33 (5.13, 10.74)	5.97 (4.31, 8.80)	**<0.001**
PBDE47	109.30 (78.97, 172.80)	124.90 (87.95, 194.20)	98.53 (72.13, 158.00)	**<0.001**
PBDE85	2.02 (1.36, 3.54)	2.34 (1.51, 3.81)	1.87 (1.32, 3.18)	**<0.001**
PBDE99	20.01 (13.94, 33.60)	23.20 (15.05, 37.53)	18.64 (13.12, 30.91)	**<0.001**
PBDE100	22.06 (15.52, 33.78)	23.86 (16.96, 38.20)	20.390 (14.73, 32.44)	**<0.001**
PBDE153	59.03 (36.79, 93.90)	59.40 (37.22, 99.81)	58.180 (36.19, 91.75)	0.35
PBDE154	1.95 (1.30, 3.20)	2.15 (1.46, 3.50)	1.73 (1.19, 2.83)	**<0.001**
PBDE209	14.79 (10.87, 20.22)	15.23 (11.47, 21.31)	13.87 (10.26, 19.32)	**<0.001**
PBB153	17.36 (10.59, 29.42)	20.11 (12.62, 35.01)	14.59 (8.94, 25.25)	**<0.001**

Bold indicates statistical significance – *p* value.

### 3.2 Association between single BFR and periodontitis

In the fully adjusted model, several ln-transformed BFR components were positively correlated with periodontitis, including serum PBDE28 (OR: 1.79; 95% CI: 1.43–2.23; *p* < 0.001), PBDE47 (OR: 1.50; 95% CI: 1.22–1.84; *p* < 0.001), PBDE85 (OR: 1.28; 95% CI: 1.09–1.50; *p* = 0.004), PBDE99 (OR: 1.34; 95% CI: 1.13–1.58; *p* < 0.001), PBDE100 (OR: 1.30; 95% CI: 1.07–1.59; *p* = 0.010), PBDE154 (OR: 1.33; 95% CI: 1.21–1.58; *p* = 0.003), PBB153 (OR: 1.45; 95% CI: 1.22–1.72; *p* < 0.001). When expressed in quartiles, PBDE28 (P for trend = 0.025), PBDE85 (P for trend = 0.011), PBDE100 (P for trend = 0.009), PBDE154 (P for trend = 0.019), and PBB153 (P for trend = 0.011) displayed an increased OR with periodontitis. The relationship between the BFR components and periodontitis is shown in Table 2.

**Table 2:** Association between brominated flame retardants components and periodontitis.

	Model 1		Model 2		Model 3	
	OR (95%CI)	*p* value	OR (95%CI)	*p* value	OR (95%CI)	*p* value
PBDE28						
Continuous	2.03 (1.65, 2.49)	**<0.001**	1.84 (1.48, 2.29)	**<0.001**	1.79 (1.43, 2.23)	**<0.001**
Quartile 1	Ref.		Ref.		Ref.	
Quartile 2	1.41 (1.01, 1.97)	**0.042**	1.46 (1.05, 2.02)	**0.026**	1.43 (1.03, 2.00)	**0.036**
Quartile 3	2.01 (1.48, 2.73)	**<0.001**	1.82 (1.31, 2.54)	**<0.001**	1.76 (1.25, 2.47)	**0.002**
Quartile 4	2.63 (1.83, 3.77)	**<0.001**	2.33 (1.62, 3.37)	**<0.001**	2.42 (1.56, 3.24)	**<0.001**
P for trend	**0.028**		**0.017**		**0.025**	
PBDE47						
Continuous	1.72 (1.42, 2.07)	**<0.001**	1.51 (1.23, 1.85)	**<0.001**	1.50 (1.22, 1.84)	**<0.001**
Quartile 1	Ref.		Ref.		Ref.	
Quartile 2	1.49 (1.16, 1.91)	**0.002**	1.30 (1.00, 1.71)	0.053	1.30 (0.99, 1.72)	0.059
Quartile 3	2.38 (1.73, 3.27)	**<0.001**	2.00 (1.43, 2.79)	**<0.001**	1.97 (1.41, 2.75)	**<0.001**
Quartile 4	2.25 (1.63, 3.10)	**<0.001**	1.80 (1.26, 2.55)	**0.002**	1.78 (1.25, 2.53)	**0.003**
P for trend	**0.003**		0.089		0.095	
PBDE85						
Continuous	1.45 (1.25, 1.69)	**<0.001**	1.28 (1.10, 1.50)	**0.003**	1.28 (1.09, 1.50)	**0.004**
Quartile 1	Ref.		Ref.		Ref.	
Quartile 2	1.54 (1.21, 1.96)	**<0.001**	1.40 (1.08, 1.82)	**0.012**	1.40 (1.07, 1.82)	**0.015**
Quartile 3	1.94 (1.44, 2.60)	**<0.001**	1.51 (1.09, 2.07)	**0.014**	1.49 (1.07, 2.08)	**0.020**
Quartile 4	1.87 (1.38, 2.55)	**<0.001**	1.47 (1.03, 2.11)	**0.036**	1.47 (1.02, 2.11)	**0.040**
P for trend	**<0.001**		**0.008**		**0.011**	
PBDE99						
Continuous	1.50 (1.30, 1.74)	**<0.001**	1.34 (1.14, 1.56)	**<0.001**	1.34 (1.13, 1.58)	**<0.001**
Quartile 1	Ref.		Ref.		Ref.	
Quartile 2	1.31 (0.99, 1.73)	0.058	1.10 (0.80, 1.5)	0.542	1.11 (0.80, 1.54)	0.530
Quartile 3	1.92 (1.45, 2.54)	**<0.001**	1.57 (1.14, 2.15)	**0.007**	1.57 (1.15, 2.14)	**0.007**
Quartile 4	2.09 (1.54, 2.83)	**<0.001**	1.66 (1.18, 2.34)	**0.005**	1.66 (1.18, 2.34)	**0.006**
P for trend	0.051		0.516		0.503	
PBDE100						
Continuous	1.52 (1.27, 1.82)	**<0.001**	1.32 (1.09, 1.60)	**0.007**	1.30 (1.07, 1.59)	**0.010**
Quartile 1	Ref.		Ref.		Ref.	
Quartile 2	1.85 (1.44, 2.56)	**<0.001**	1.70 (0.20, 2.40)	**0.004**	1.65 (1.16, 2.35)	**0.008**
Quartile 3	1.98 (1.44, 2.73)	**<0.001**	1.59 (1.16, 2.18)	**0.005**	1.57 (1.14, 2.16)	**0.008**
Quartile 4	2.05 (1.47, 2.85)	**<0.001**	1.63 (1.14, 2.33)	**0.010**	1.59 (1.11, 2.29)	**0.014**
P for trend	**<0.001**		**0.005**		**0.009**	
PBDE153						
Continuous	1.10 (0.94, 1.28)	0.218	1.01 (0.83, 1.23)	0.887	1.01 (0.83, 1.23)	0.925
Quartile 1	Ref.		Ref.		Ref.	
Quartile 2	0.88 (0.63, 1.21)	0.413	0.74 (0.53, 1.04)	0.080	0.75 (0.53, 1.05)	0.087
Quartile 3	0.98 (0.69, 1.38)	0.892	0.88 (0.61, 1.27)	0.496	0.87 (0.60, 1.27)	0.469
Quartile 4	0.99 (0.75, 1.29)	0.987	0.81 (0.57, 1.13)	0.197	0.81 (0.58, 1.13)	0.202
P for trend	0.755		0.541		0.516	
PBDE154						
Continuous	1.53 (1.30, 1.80)	**<0.001**	1.22 (1.12, 1.59)	**0.002**	1.33 (1.12, 1.58)	**0.003**
Quartile 1	Ref.		Ref.		Ref.	
Quartile 2	1.55 (1.15, 2.07)	**0.004**	1.59 (1.14, 2.20)	**0.008**	1.56 (1.12, 2.17)	**0.011**
Quartile 3	1.98 (1.41, 2.78)	**<0.001**	1.61 (1.10, 2.35)	**0.016**	1.60 (1.09, 2.35)	**0.019**
Quartile 4	2.00 (1.45, 2.74)	**<0.001**	1.62 (1.15, 2.29)	**0.007**	1.60 (1.14, 2.25)	**0.009**
P for trend	**0.008**		**0.014**		**0.019**	
PBDE209						
Continuous	1.42 (1.18, 1.71)	**<0.001**	1.15 (0.95, 1.40)	0.153	1.14 (0.95, 1.38)	0.162
Quartile 1	Ref.		Ref.		Ref.	
Quartile 2	1.38 (1.05, 1.82)	**0.024**	1.35 (1.01, 1.80)	**0.045**	1.33 (0.99, 1.79)	0.057
Quartile 3	1.66 (1.25, 2.21)	**<0.001**	1.35 (1.00, 1.82)	**0.047**	1.33 (0.98, 1.80)	0.063
Quartile 4	1.65 (1.28, 2.12)	**<0.001**	1.27 (0.98, 1.64)	0.070	1.26 (0.98, 1.61)	0.072
P for trend	0.478		0.813		0.790	
PBB153						
Continuous	1.41 (1.23, 1.61)	**<0.001**	1.48 (1.25, 1.76)	**<0.001**	1.45 (1.22, 1.72)	**<0.001**
Quartile 1	Ref.		Ref.		Ref.	
Quartile 2	1.69 (1.15, 2.46)	**0.008**	2.31 (1.57, 3.41)	**<0.001**	2.24 (1.51, 3.34)	**<0.001**
Quartile 3	2.54 (1.77, 3.65)	**<0.001**	3.30 (2.16, 5.04)	**<0.001**	3.13 (2.03, 4.83)	**<0.001**
Quartile 4	2.79 (1.93, 4.05)	**<0.001**	3.39 (2.16, 5.33)	**<0.001**	3.19 (2.00, 5.10)	**<0.001**
P for trend	**<0.001**		**0.004**		**0.011**	

Model 1 was unadjusted.

Model 2 was adjusted for age, gender, race, poverty-to-income ratio, education levels, body mass index, energy intake, smoking status and alcohol use.

Model 3 was adjusted for age, gender, race, poverty-to-income ratio, education levels, body mass index, energy intake, smoking status, alcohol use, sleep trouble, hypertension, diabetes and hyperlipidemia.

Bold indicates *p* value < 0.05. OR, odds ratio; CI, confidence interval.

### 3.3 Analysis of restricted cubic spline regression

**Figure 2:**
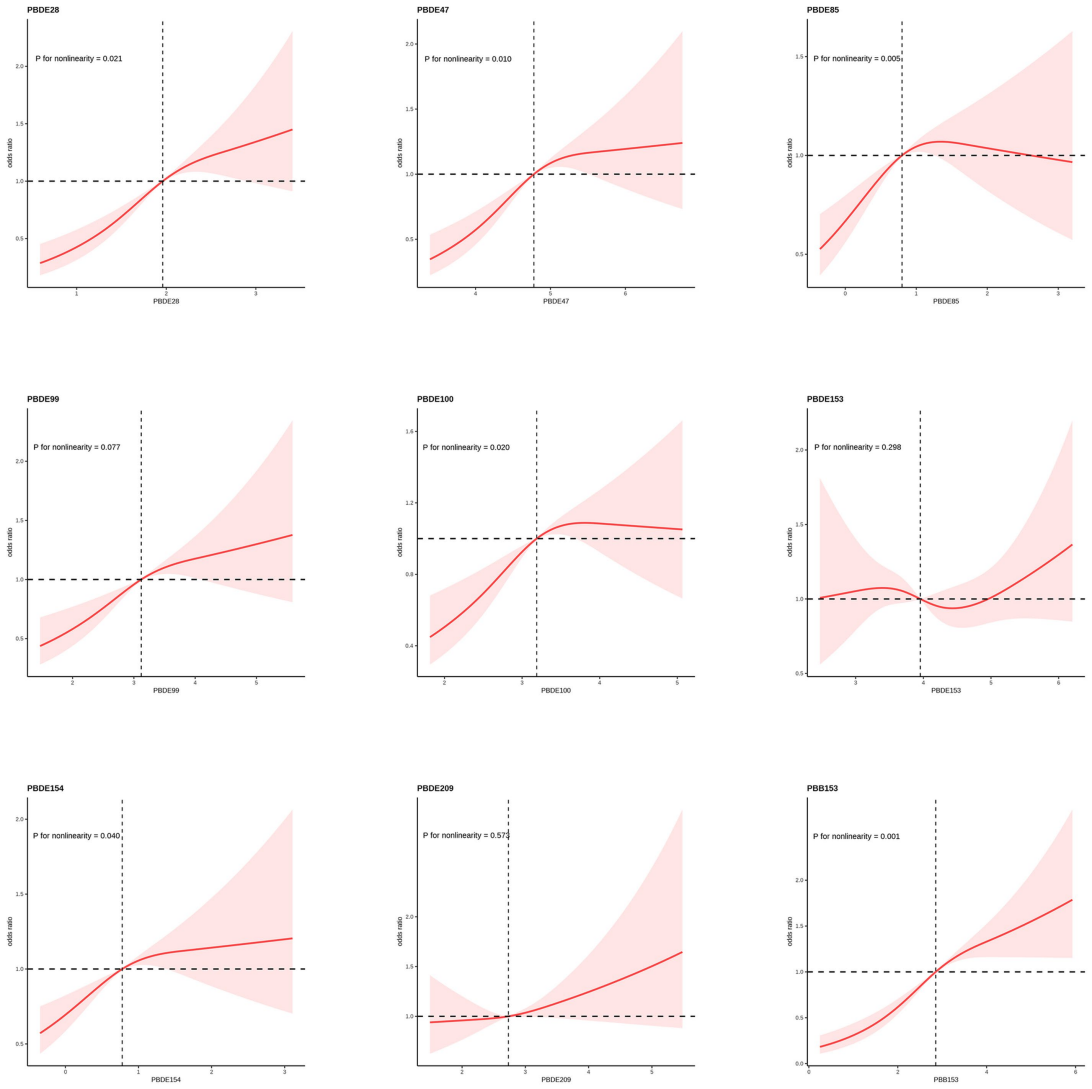
Nonlinear correlation between BFR components and periodontitis in RCS regression.

An analysis of the RCS regression is shown in Figure 2. After adjusting for all covariates, we found a significant nonlinear correlation between ln-transformed PBDE28 (*p* = 0.021), PBED47 (*p* = 0.010), PBDE85 (*p* = 0.005), PBDE100 (*p* = 0.020), PBDE154 (*p* = 0.040) and PBB153 (*p* = 0.001) with periodontitis in the RCS regression. Among them, PBDE85 exhibited a distinctive inverted U-shaped association, while PBDE47, PBDE154, and PBDE100 showed a more plateau-like trend.

### 3.4 The combined effects of BFRs on periodontitis

**Figure 3:**
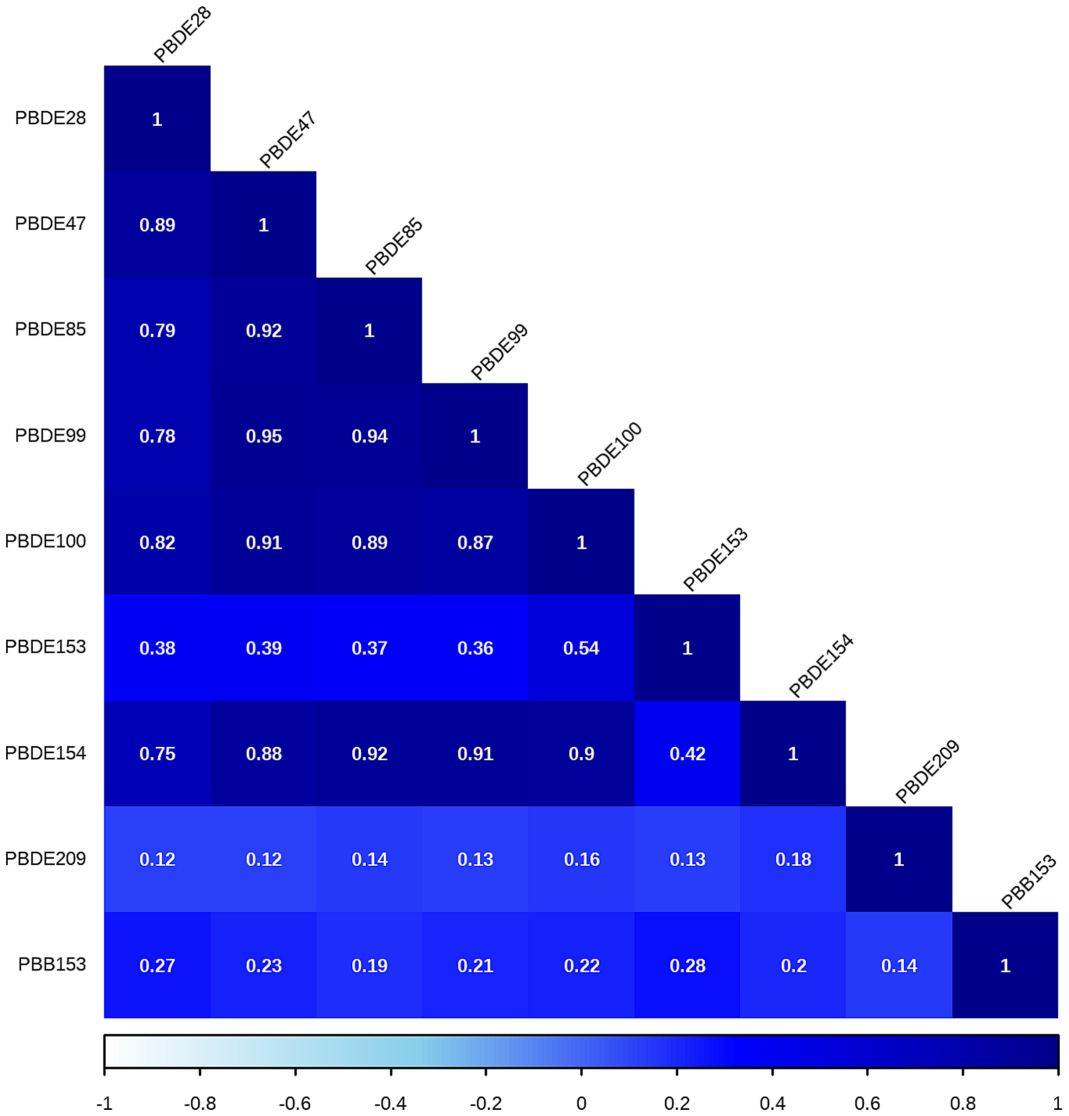
Spearman’s rank correlation coefficients between BFR components.

**Figure 4:**
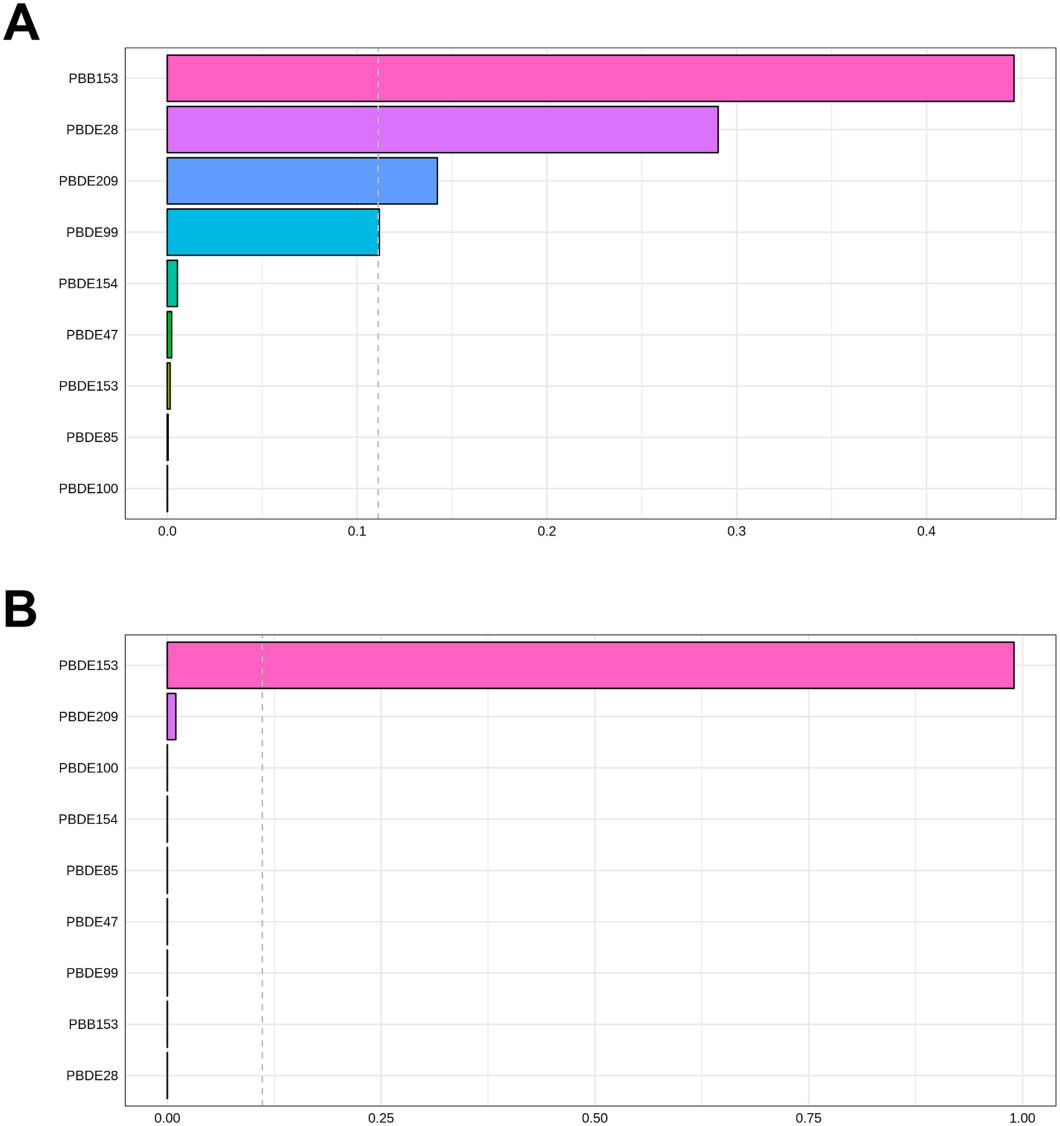
Association of BFRs mixture in WQS regression with periodontitis. **(A)** Positive correlation analysis. **(B)** Negative correlation analysis.

**Figure 5:**
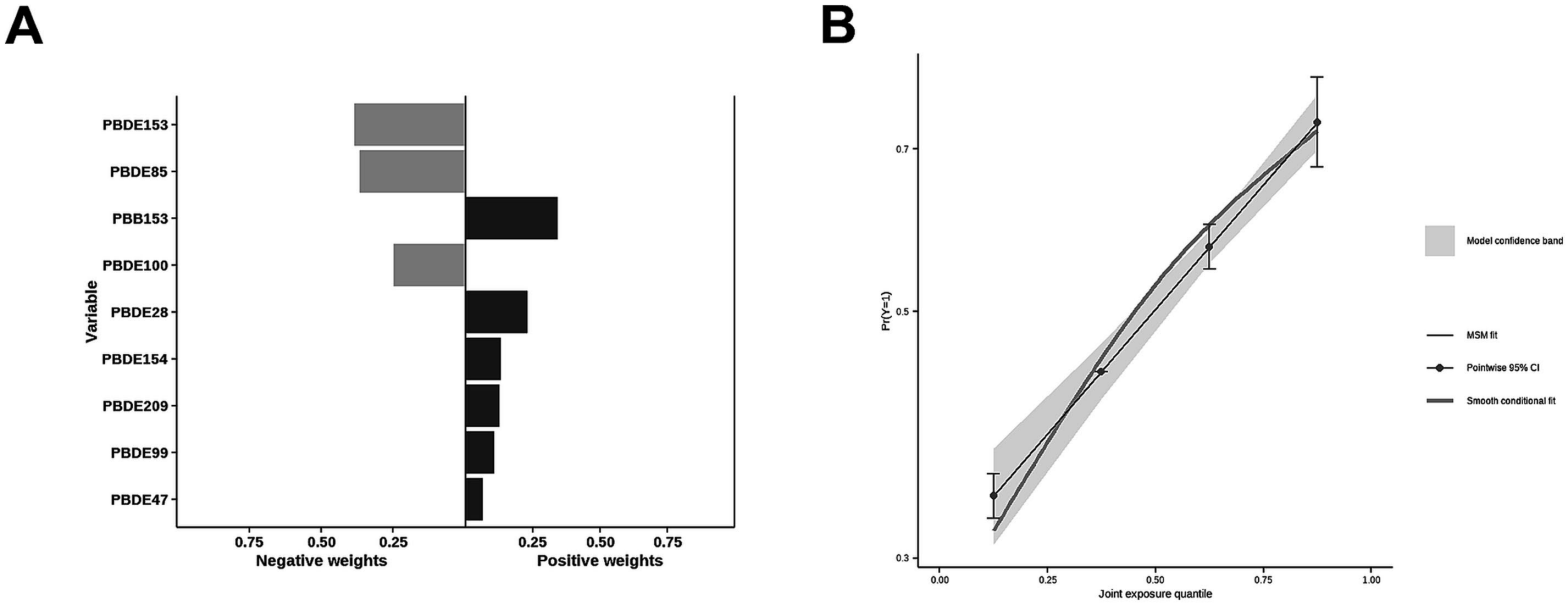
Association of BFRs mixture in QGC analysis with Periodontitis. **(A)** Proportion for Periodontitis. **(B)** Combined effects for Periodontitis.

The correlations between each pair of ln-transformed BFRs, as indicated by the Spearman’s rank correlation coefficient, are presented in Figure 3. Among these components, PBDE47, PBDE85, PBDE99, PBDE100, and PBDE154 exhibited strong correlations with each other. The analysis using WQS regression and QGC analysis found a positive association between the exposure to mixture BFRs and the prevalence of periodontitis. The WQS index of mixture BFRs exposure was positively associated with periodontitis prevalence (OR: 1.52; 95% CI: 1.30–1.79, *p* < 0.001), with PBB153, PBDE28, PBDE209 and PBDE99 having a relatively stronger impact (Figure 4). However, no significant association was observed when analyzing in the negative direction (OR: 0.91; 95% CI: 0.82, 1.02; *p* = 0.10). In the QGC analysis, exposure to mixture BFRs also demonstrated similar outcomes, showing a positive association with the prevalence of periodontitis (QGC *β*: 1.29; 95% CI: 1.24–1.36, *p* < 0.001). Within the serum BFRs, PBB153, PBDE28, PBDE154, PBDE209, PBDE99, and PBDE47 exhibited positive weights, while PBDE153, PBDE85, and PBDE100 displayed negative weights (Figure 5).

## 4 Discussion

Based on population-representative data, we have found that despite several BFRs being withdrawn from the U.S. market years ago, their concentrations in human serum remain high. Building on this foundation, this study is the first to investigate the correlation between BFRs and periodontitis. The results indicate that the quartile concentrations of PBDE28, PBDE85, PBDE100, PBDE154, and PBB153 are positively associated with the incidence of periodontitis. Additionally, a non-linear relationship was observed between the concentrations of PBDE28, PBDE47, PBDE85, PBDE100, PBDE154, PBB153, and the occurrence of periodontitis. Findings from WQS regression and QGC analyses further suggest that mixed BFR exposure is positively associated with periodontitis. These compelling findings provide evidence of the detrimental impact of BFR exposure on periodontal health and underscore the importance of further research into the potential health consequences of these compounds.

Currently, there is a lack of research on the relationship between BFRs and periodontitis. The positive correlation observed in this study between BFR exposure and the prevalence of periodontitis may be linked to factors such as oxidative stress, immune responses, and bone metabolism.

The imbalance between prooxidants and antioxidants can lead to oxidative stress, characterized by an increase in reactive oxygen species (ROS) production (28). Recently, there has been growing recognition of the role of BFR in inducing oxidative stress. PBDE and PBB can induce oxidative stress through various pathways, including disrupting the metabolic homeostasis of HepG2 cells, activating the Nrf2-mediated antioxidant response and P38 MAPK pathway, and signaling through the IRE1a/AKT/mTOR pathway (29–31). The antioxidant system plays a crucial role in protecting cells from oxidative stress and harmful exogenous compounds by neutralizing free radicals. Several BFR compounds significantly reduce the activities of antioxidant enzymes such as superoxide dismutase (SOD), catalase (CAT), glutathione peroxidase (GSH-Px), and reduced glutathione (GSH), further exacerbating ROS generation (32). Prolonged and excessive ROS production leads to elevated levels of immune cell factors, triggering a cascade of signaling reactions that can cause decoupling of bone remodeling in periodontitis (33). Multiple studies have substantiated a clear link between the levels of oxidative stress markers or antioxidants and the occurrence, severity, or amelioration of periodontitis (34).

Inflammation is defined by the activation of diverse immune cells in both the innate and adaptive immune systems, leading to increased production of immune cytokines in the cellular milieu (35). Both animal and human studies have demonstrated that exposure to BFRs results in increased secretion of pro-inflammatory cytokines such as IL-1β, IL-6, and TNF-*α* (36–38). Simultaneously, BFR reduces the expression of anti-inflammatory cytokines such as IL-4, IL-10, and IL-13 through pathways including PI3 kinase, AP-1 and NF-kappaB (38). In the development of periodontitis, the initiation of immune and inflammatory responses plays a crucial role (13). NF-kappaB is a particularly important signaling pathway in this process, regulating the proliferation and differentiation of macrophages and lymphocytes (35).

Animal studies have demonstrated that in primary bone marrow cultures of mice, BFR exposure can activate PPARγ1 and 2 and suppress osteogenesis, as indicated by reduced alkaline phosphatase activity and Osx expression (39). A cross-sectional study based on American adults showed that serum BFR levels negatively predicted bone mineral density (BMD) in men (19). The correlation between periodontitis and BMD was initially identified in the 1960s. A 2017 workshop concluded that osteoporosis is significantly linked to an increased prevalence and severity of radiographic alveolar bone loss. At present, a substantial body of research and systematic reviews supports the connection between BMD and CAL or other clinical indicators of periodontitis (33).

In this study, the dose–response patterns observed between BFR and periodontitis prevalence vary, predominantly displaying either a plateau or an inverted U-shaped relationship. Previous research indicates that as endocrine-disrupting chemicals, BFRs do not always follow the traditional toxicological monotonic dose–response relationship. Instead, they may exhibit different dose–response curves under varying exposure distributions. Receptor-mediated responses can increase with the dose initially and then decrease, forming an inverted U-shaped curve (40). Additionally, receptor-mediated responses typically show strong dose-dependence, followed by a plateau phase where the response ceases to increase with further dose escalation, resulting in a plateau-shaped curve (41).

Most BFRs appear to be non-toxic; however, the complexity of BFRs in the environment presents challenges for research, raising concerns that these compounds or potential contaminants within BFR mixtures may interact with cells (42). According to the results of Spearman’s rank correlation coefficient in Figure 3, we observe collinearity among the chemical substances in BFRs. Under such conditions, traditional analyses that model a single chemical at a time may lead to biased results. This necessitates the adoption of new approaches to manage multicollinearity and high-dimensional data challenges (43, 44). Previous studies have found significant differences in the toxicity of BFR mixtures compared to individual components, potentially exhibiting antagonistic or synergistic effects, which traditional regression models struggle to accurately estimate (6, 21, 22). Therefore, this study utilized robust statistical methods, including WQS regression and QGC models, to explore the potential impacts of serum BFR mixtures on periodontitis. The results from both models exhibit consistent findings. There is a significant positive association between BFR mixtures and periodontitis. Among the chemicals, PBB153 holds the highest weight, which is consistent with the results from the individual component analysis. This may be attributed to the fact that PBB153 has a relatively long half-life in the human body, posing a higher health risk to humans (22). Through these advanced methods, this study provides more comprehensive evidence, revealing the potential health impacts of BFR mixtures on periodontitis.

### 4.1 Strengths

Firstly, the data were derived from a nationally representative sample of adults in the United States, enhancing the generalizability and applicability of the findings. Secondly, the study not only analyzed the relationship between individual BFR components and periodontitis but also examined the impact of BFR mixtures, providing a more comprehensive perspective. Thirdly, complex statistical models were developed to control for confounding factors as much as possible, thereby increasing the reliability and scientific validity of the conclusions.

### 4.2 Limitations

Firstly, as a cross-sectional study, it cannot establish a causal relationship between BFR exposure and periodontitis. Secondly, due to the lack of relevant data in the NHANES dataset, this study did not include newer BFRs developed as replacements for PBDEs. Thirdly, although we made efforts to control for confounding variables, there may still be unaccounted factors influencing the results. Therefore, further research is needed to improve control of potential confounders, and large-scale longitudinal studies are necessary to better elucidate the causal relationship between BFR exposure and periodontitis.

## 5 Conclusion

In conclusion, this study shows a positive association between BFR exposure and periodontitis. Significant correlations were found with several BFR components, and both WQS and QGC analyses confirmed increased prevalence of periodontitis with mixed BFR exposure. Our findings will help raise public awareness about preventing BFR exposure and promote efforts to find safer alternatives to BFRs for human health.

## Data Availability

Publicly available datasets were analyzed in this study. This data can be found here: https://wwwn.cdc.gov/nchs/nhanes/.

## Footnotes

^1^ https://www.cdc.gov/nchs/nhanes
